# *Schistosoma hematobium* and *S. mansoni* among Children, Southern Sudan

**DOI:** 10.3201/eid1310.070356

**Published:** 2007-10

**Authors:** Roberto Deganello, Mario Cruciani, Claudio Beltramello, Otine Duncan, Vincent Oyugi, Antonio Montresor

**Affiliations:** * World Health Organization Headquarters for Southern Sudan, Nairobi, Kenya; † Center of Preventive Medicine and HIV Outpatient Clinic, Verona, Italy; ‡Ospedale di Treviso, Treviso, Italy; § Comitato di Coordinamento delle Organizzazioni per il Servizio Volontario, Southern Sudan, Geneva, Switzerland; and ¶World Health Organization, Hanoi, Vietnam; 1 Current affiliation: University of Verona, Verona, Italy

**Keywords:** Schistosomiasis, Schistosoma mansoni, Schistosoma haematobium, prevalence, Sudan, epidemiology, dispatch

## Abstract

We conducted a survey of schistosomiasis among schoolchildren in 2 villages in Southern Sudan. In Lui (West Equatoria region), prevalence of *Schistosoma mansoni* infection was 51.5%; no cases of *S. hematobium* infection were detected. In Nyal (Upper Nile region), prevalence of *S. hematobium* infection was 73% and *S. mansoni* infection, 70%.

Schistosomiasis is a major communicable disease of public health and socioeconomic importance in the developing world. Both *Schistosoma hematobium* and *S. mansoni* are present in Sudan, a war-torn country with a population of ≈6 million persons and one of the world’s most underdeveloped regions. Risk for schistosomiasis in Sudan is widespread, especially in the major irrigation systems in the Gezira area between the Blue and White Nile Rivers.

Early reports stated that schistosomiasis is endemic in southern provinces, but few sustained surveys have been conducted, particularly during the years overwhelmed by the civil war, and recent data on prevalence rates are lacking (*1*–*3*,*5*–*10*). We therefore conducted an epidemiologic survey in 2 villages in Southern Sudan.

## The Study

This investigation was a cross-sectional study conducted in 2 separate regions of Southern Sudan from August through October 2002 (rainy season). The study population was recruited from the only primary schools in the village of Lui (Mundri County), West Equatoria Region, and from 3 randomly selected primary schools in the district of Nyal (for a total of 4 schools), Upper Nile Region, 40 km west of the Nile River (Figure). In 2006, an estimated 740,000 persons lived in the West Equatoria region, while an estimated 221,667 persons lived in Mundri County. An estimated 11,500–15,000 persons lived in Nyal village. The ecologic situation (rain, presence of water bodies, population density) and the presence of sanitation structures are homogenously distributed in Nyal District, and the populations of the schools we selected were representative of the parasitologic situation in this area. Lui has an estimated population of 7,000–9,000 persons and a primary school only.

**Figure:**
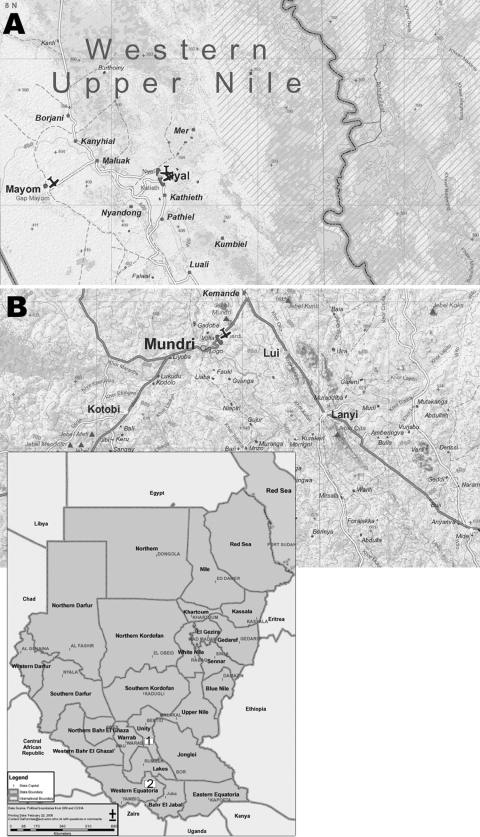
Location of the study areas in Southern Sudan: A) Western Upper Nile; B) West Equatoria Region, Mundri County (source: Centre for Development and Environment, University of Berne, Switzerland; available from www.cde.unibe.ch/sudan/maps), with an inset of the whole country (source: World Health Organization; available from www.emro.who.int/sudan/media/pdf/sud-states-2006.pdf).

To assess the prevalence of schistosomiasis, we conducted a survey of stool and urine samples as recommended by the World Health Organization (WHO) (*11*). A sample of 200–250 children in each ecologically homogenous area was considered adequate to evaluate prevalence and intensity of *Schistosoma* infection in a disease-endemic area. Before the onset of the study, information meetings were held with the school staff. Schoolchildren were asked to provide a stool sample and at least 10 mL of urine. Consent was obtained directly from the children (or parents or teachers) before specimen collection. The study was part of the routine investigations conducted by the Sudan Ministry of Health and WHO to provide adequate treatment to the children in the area. Because the surveyed children were not exposed to any risk (they only provided stool and urine samples), no special approval was requested.

Specimens were collected at school and brought to the laboratory, where they were prepared and interpreted within 24 hours. This procedure was repeated over 2 days in each village surveyed. Urine samples were tested for *S. hematobium* infection by using the Urine Filtration Kit (Vestergaard-Frandsen, Nairobi, Kenya) provided by WHO. Fecal specimens were tested for *S. mansoni* infection by using the Kato Katz semiquantitative method (*11*,*12*). Infections were classified as high intensity if results were >50 eggs of *S. hematobium* per 10 mL of urine or at least 400 eggs of *S. mansoni* per gram of feces. Because, for technical reasons, urine and stool specimens were collected on separate days, we have no data on dual *S. hematobium* and *S. mansoni* infection.

Urine specimens were obtained from 400 children, 200 in each village; fecal specimens were obtained from 400 children, 200 in each village. The age range was 8–12 years; 75% of those who provided samples were boys. The disparity between the sexes is probably related to more unwillingness of the girls to provide urine or fecal specimens.

The prevalence and intensity of *S. hematobium* and of *S. mansoni,* by geographic area, are shown in the Table. Though the prevalence was higher for girls than for boys, these differences were not statistically significant by χ^2^ test (data not shown). As a consequence of the high prevalence of schistosomiasis, praziquantel (40 mg/kg) treatment was offered to all children in the surveyed schools.

**Table:** Prevalence and intensity of *Schistosoma mansoni* and *S. hematobium* in 2 villages in Southern Sudan

Location	*S. hematobium*			*S. mansoni*		
	No. positive specimens/total examined (%)	No. high intensity infections (%)*		No. positive specimens/total examined (%)	No. moderate intensity infections (%)†	No. heavy intensity infections (%)‡
Lui (West Equatoria)	0/200	0		103/200 (51.5)	33 (16.5)	16 (8)
Nyal (Upper Nile)	146/200 (73)	57 (28.5)		140/200 (70)	54 (27)	37 (18.5)

*>50 eggs/10 mL urine. †100–399 eggs/g feces. ‡>400 eggs/g feces.

## Conclusions

Most epidemiologic studies regarding schistosomiasis in Sudan have been carried out in the Gezira-Managil area and in other central or northern areas of economic importance, while relatively few studies have been conducted in other parts of the country. Our study was conducted in 2 ecologically different areas in Southern Sudan. The West Equatoria region is a savannah area, topographically and ethnographically homogeneous with the bordering regions of Uganda, characterized by a prevalence of intestinal bilharziasis (*8*). The Upper Nile region is a swampy area and has been considered by the Sudanese authorities as being nonendemic for bilharziasis (*3*,*8*).

The long-running civil war has overshadowed Southern Sudan’s recent history and devastated social and health services. Updated epidemiologic data are thus lacking, and most epidemiologic reports from these areas are scattered, incomplete, and outdated (1930–1970) (*1*–*10*). Data from annual reports of the Sudan Medical service in 1939–1949 and from other published or unpublished sources indicated that *S. mansoni* and S. *hematobium* were found in 1.9% and 2.5% of specimens in the Upper Nile region and in 4.9% and 0.25% of specimens in the Bahr El Ghazal region (close to the Northern Sudan border); in the West Equatoria region, the prevalence of *S. mansoni–*positive specimens has been reported as high as 44.3%, but no specimen was positive for *S. hematobium* (*3*,*8*). These data were based mainly on routine examination of urine and stools in hospitals and dispensaries. Subsequently, Amin and Omer suggested a 4.8%–6.8% prevalence of *S. mansoni* infection and a 0.4%–44% prevalence of *S. hematobium* infection in different areas of Southern Sudan (*9*).

Since the 1970s, few observations on the prevalence of schistosomiasis in southern Sudan have been made. Magambo et al. in 1998 reported a *S. mansoni* prevalence of 2.2% among children in 2 primary schools in the East Equatoria region, very close to the Ugandan border (*10*). A report conducted in the early 1990s in Juba (the largest and most developed town in Southern Sudan under the Northern government control) showed that 66% of refugees were harboring intestinal helminths, and in 26% of cases, *S. mansoni* (*13*). Data from Rhino camp, one of the major Ugandan camps accommodating Southern Sudanese refugees, showed a prevalence of *S. mansoni* as high as 77.8% (*14*). This potential impact of population mobility on the transmission of schistosomiasis should be taken into consideration. Actually, the January 2005 peace agreement between Khartoum and the Sudan People’s Liberation Movement is expected to trigger the return of half a million Sudanese who fled to nearby countries and the gradual resettlement of millions internally displaced. Increased efforts by the international community to help meet the basic needs of this rapidly growing population are urgently needed.

Our data confirm a high prevalence of *S. mansoni* infection in some areas in the West Equatoria region and show that both *S. mansoni* and *S. hematobium* are highly endemic in the Upper Nile region. The collected data facilitated schistosomiasis control interventions in the area. Mass distribution of praziquantel to all schoolchildren has been organized in the 2 districts since 2003.

When one considers the extremely high prevalence and the very low cost of the control measures (*15*), mass administration of praziquantel should be annually provided to the children in the resident population and to the children in the displaced population that will resettle to these areas as a consequence of peace agreements. These data also provide further evidence supporting the Centers for Disease Control and Prevention recommendation (available from www.cdc.gov/ncidod/dq/pdf/lost%20boys%20and%20girls%20presumptive%20treatment%20recommendations.pdf) that members of the Lost Boys and Girls of Sudan refugee group who have resettled in the United States and other Sudanese refugees receive presumptive therapy for schistosomiasis.
